# Zinc and Traumatic Brain Injury: From Chelation to Supplementation

**DOI:** 10.3390/medsci8030036

**Published:** 2020-08-17

**Authors:** Cathy W. Levenson

**Affiliations:** Department of Biomedical Sciences and Program in Neuroscience, College of Medicine, Florida State University, Tallahassee, FL 32306, USA; cathy.levenson@med.fsu.edu

**Keywords:** brain injury, trauma, zinc, excitotoxicity, neuroprotection, chelation, supplementation

## Abstract

With a worldwide incidence rate of almost 70 million annually, traumatic brain injury (TBI) is a frequent cause of both disability and death. Our modern understanding of the zinc-regulated neurochemical, cellular, and molecular mechanisms associated with TBI is the result of a continuum of research spanning more than three decades. This review describes the evolution of the field beginning with the initial landmark work on the toxicity of excess neuronal zinc accumulation after injury. It further shows how the field has expanded and shifted to include examination of the cellular pools of zinc after TBI, identification of the role of zinc in TBI-regulated gene expression and neurogenesis, and the use of zinc to prevent cognitive and behavioral deficits associated with brain injury.

## 1. Introduction

A recent meta-analysis estimated the worldwide incidence of traumatic brain injury (TBI) to be approximately 69 million annually. Of these, 56 million appear to be mild cases (mTBI), with the remainder being moderate and severe [1]. Patients can develop behavioral deficits including impairments in attention, memory, and executive function. Anxiety, aggression, poor social functioning, and posttraumatic stress disorder (PTSD) have all also been reported following TBI [2]. The most common consequence of TBI, major depression, is reported in all forms of TBI, including mTBI, and affects as many as 40% of individuals with brain injury [3,4].

Early work revealed the toxic nature of zinc accumulation in brain regions such as the hippocampus and cortex following seizure, ischemia, and TBI [5,6]. This work showed that excess zinc leads to postsynaptic neuronal death. While zinc clearly plays a deadly role in the acute neuronal response to injury, it is also needed for longer-term reparative processes following injury including gene expression and neurogenesis. Thus, while there have been many studies showing that chelation of zinc following neuronal injury can provide neuroprotection after injury, there has been a recent trend to examine the role that appropriately timed zinc supplementation may play in preventing poor outcomes associated with TBI.

This review explores the continuum of TBI research that led investigators to first examine the potential benefits of zinc chelation and then move on to the possible neuroprotective roles of zinc and an exploration of zinc supplementation to improve outcomes associated with TBI.

## 2. TBI and Zinc Toxicity

Recent studies show that TBI does not change total brain zinc levels [7]. This is consistent with data showing that the damage caused by zinc after TBI is the result of rapid shifts in zinc localization that result in damage to neurons and other cells [6,8]. In zinc-rich regions of the brain, including the hippocampus, cerebral cortex, amygdala, olfactory bulb, and cingulate cortex, as much as 30% of the zinc is sequestered in neuronal vesicles. Upon excitation, presynaptic neurons release vesicular zinc into the synaptic cleft between neurons where it binds and modulates a variety of postsynaptic receptors and channels such as the glutamate receptors *N*-methyl-d-aspartate (NMDA), α-amino-3-hydroxy-5-methyl-4-isoxazolepropionic acid (AMPA), and kainate receptors, as well as glycine, γ-aminobutyric acid-A (GABA_A_) receptors, and voltage-gated calcium channels [9]. This relationship led to work suggesting that TBI and other forms of injury induce the rapid release of excess zinc from presynaptic neuronal vesicles resulting in postsynaptic accumulation of toxic levels of zinc, calcium influx, excitotoxicity, and neuronal death [6,8,10].

### 2.1. Zinc Sources

While the source of the toxic zinc was first thought to be vesicular, subsequent work showed that vesicular zinc was not essential for neuronal damage [11]. Specifically, investigators used transgenic animals that lacked vesicular zinc (*ZnT-3* null mice). When seizures were induced in these animals, zinc accumulated in damaged neurons in the hippocampus showing that there are clearly other pools of zinc that translocate, accumulate, and contribute to neuronal death. These data led to a re-evaluation of the role of vesicular zinc [12,13] and were followed by convincing in vivo work showing that vesicular zinc may, in fact, play no role in the translocation and accumulation of excess free zinc after TBI [14].

If the source of excess zinc in TBI is not vesicular, as was so long believed, what then, are the other sources? While the answer to this question is still incomplete, a leading contender is the zinc-binding family of metallothioneins, especially the brain-specific metallothionein-3 (MT-3). Early work showed that TBI resulted in increased *MT-3* gene expression [15]. Furthermore, zinc accumulation and neuronal death were both significantly decreased after seizure in *MT-3* null mice in the hippocampus (CA1), dentate gyrus, and thalamus [16,17]. Another potential source of excess neuronal zinc following TBI is mitochondrial zinc stores [18,19].

### 2.2. Mechanisms of Toxicity

Regardless of the exact sources of excess zinc after injury, it is clear that acute increases in cytosolic zinc are neurotoxic. There is a long list of potential mechanisms whereby excess zinc acts to induce neuronal damage and death. For example, it has been hypothesized that release of zinc from MT-3 after injury induces reactive oxygen species [20]. The resulting oxidative stress then leads to a cascade of secondary injury mechanisms such as mitochondrial disruption and inflammation [21]. Other mechanisms that appear to be involved include zinc-induced NAD^+^ deficiency that impairs glycolysis [22,23], activation of the nerve growth factor receptor TrkA [24], zinc-mediated mitochondrial dysfunction [25], and poly(ADP-ribose) polymerase (PARP) activation [26]. Excess zinc also induced ubiquitin tagging of neuronal proteins for destruction. Scavenging of zinc after TBI reduced ubiquitin conjugation and protected hippocampal neurons [27]. Together, these mechanisms lead to hybrid forms of cell death that have characteristics of both apoptosis and necrosis [28], as well as neuronal death by autophagy [29].

## 3. TBI and Zinc Restriction

### 3.1. TBI and Zinc Chelation

Recognition of the possible role of excess free zinc in the pathology associated with TBI and other forms of brain injury led to the exploration of zinc chelation as an approach to the prevention of injury-induced neuronal death. Initial studies used a seizure model of injury (kainate injection) that produced pronounced zinc accumulation and degeneration of neurons in the hippocampus. Intraventricular injection of calcium-EDTA (CaEDTA) into the brain successfully blocked cytosolic zinc accumulation and neuronal degeneration [10]. Of particular note is that CaEDTA does not cross cell membranes. This suggests that after injury, excess zinc accumulates in the extracellular space before entry into neurons where it induces damage.

Similar results have been reported for TBI. For example, in a rat fluid percussion model of TBI, intraventricular administration of CaEDTA immediately prior to injury not only provided significant neuroprotection in the CA1 region of the hippocampus, dentate, and hilus [6] but also upregulated a variety of potentially neuroprotective genes that code for proteins including heat shock proteins 27 and 70, glutathione peroxidase, and the anti-apoptotic protein p21 [30]. In the controlled cortical impact model of TBI, the zinc chelator TPEN was injected immediately after injury. This treatment prevented free zinc accumulation, reduced excitotoxicity, and improved histological markers.

While this work pointed to zinc chelation as a promising approach to improving outcomes after TBI, a shift in the field came when new work showed that chelation of zinc contributed to neuronal damage when neurons of the hippocampus were overexcited [31]. While this work was conducted in a subthreshold model of kainate-induced seizure, the fact that it suggested a protective role for synaptic zinc led to the re-examination of the role of zinc in models of TBI. Specifically, the role of zinc chelation as a treatment (not pretreatment) for TBI needed to be evaluated using functional outcomes, not just cellular, molecular, or histological markers. The next level of experiments, therefore, included functional studies of spatial learning and memory after TBI. This work showed that despite the fact that treatment of TBI with CaEDTA reduced the number of damaged neurons in the CA3 region of the hippocampus, chelation did not improve Morris water maze performance. Additionally of note was the finding that treatment with CaEDTA increased the pro-apoptotic proteins BAX and caspase-3 two weeks after injury [32], suggesting that chelation may lead to a second wave of injury weeks after the initial insult. Subsequent in vitro and in vivo work found that chelation or blocking of ionic zinc after TBI has deleterious effects in the acute period following injury (within 24 h) as well, increasing both apoptosis and necrosis in the hippocampus [14], in part by increasing reactive oxygen species [33].

### 3.2. TBI and Zinc Deficiency

As evidence that zinc chelation after brain injury could have deleterious effects continued to mount, investigators next turned their attention to the possible effects of zinc deficiency in TBI. This next phase in the research continuum was supported by early clinical observations that TBI increased urinary zinc excretion resulting in significantly depressed serum zinc levels. The most severe cases of head trauma resulted in zinc excretion rates that were over 14-fold above normal [34].

Because it is well known that TBI induces neuronal precursor proliferation and neurogenesis in the subgranular zone of the hippocampus, several investigators have examined the effect of zinc deficiency on neurogenesis. Initial studies in vitro suggested that zinc deprivation impairs neuronal precursor proliferation and induces apoptosis in these cells via p53 and caspase-mediated mechanisms [35,36,37]. However, major advances were made when investigators were able to show an essential role for zinc in adult neuronal precursor cell proliferation and neurogenesis in vivo. Dietary zinc deficiency impaired neuronal precursor cell proliferation and hippocampal neurogenesis in both rats [35,38] and mice [39]. Furthermore, neurogenesis was significantly reduced in *ZnT-3* null mice that lacked presynaptic vesicular zinc [38].

When the effects of zinc deficiency were extended to an animal model of TBI, it was shown that diets marginally deficient in zinc (5 ppm) did not significantly worsen behavioral outcomes associated with TBI including spatial learning and memory, anxiety, and depression-like behaviors [40]. However, there is evidence that marginal zinc deficiency may be linked to alterations in matrix metalloproteinases that play roles in a variety of important functions related to TBI including disruption of the blood–brain barrier, inflammation, and angiogenesis [41,42]. Furthermore, zinc chelation inhibited TBI-induced neurogenesis [43], supporting a role for vesicular and other pools of free zinc in the repair mechanisms following TBI.

## 4. TBI and Zinc Supplementation

As investigators continued to collect data suggesting important roles for zinc in the brain after injury, TBI research began to shift to studies that explored the potential use of supplemental zinc as a neuroprotective agent.

### 4.1. Clinical Trial

To test the hypothesis that zinc supplementation could improve outcomes after TBI, 68 patients with moderate-to-severe brain injuries were randomly assigned to either a zinc-adequate or zinc-supplemented treatment group for 3 months. After one month, mortality in the zinc-supplemented group was half that of the adequate control group (12% vs. 26%). Functional outcomes using the Glasgow Coma Scale were significantly improved in the zinc-supplemented patients as early as 2 weeks and continued to be evident throughout the 3 month study [44]. A recent double-blind controlled study confirmed the efficacy of zinc supplementation in patients with severe head trauma. In this trial, 100 patients with severe TBI were randomly assigned to a supplementation (120 mg zinc/day) or placebo group with assessments over 16 days. While there were no differences between the two groups at baseline, zinc supplementation resulted in significantly higher plasma zinc concentrations (*p* < 0.001), improved measures on the Sequential Organ Failure Assessment (*p* < 0.05) and Glasgow Coma Scale (*p* < 0.05), and reduced length of hospital stay (*p* < 0.05) [45].

### 4.2. Animal Studies

The use of dietary zinc supplementation in rodent models of TBI not only affords us the opportunity to examine the effect of specific behavioral and functional outcomes but also permits the study of cellular and molecular mechanisms in the brain after injury that cannot be explored in clinical trials with humans. To test the hypothesis that zinc can be used as a treatment for TBI, adult rats were subjected to a moderately severe injury by controlled cortical impact [46]. Animals were then divided into two dietary zinc treatment groups: zinc-adequate diet (control, 30 ppm) and a zinc-supplemented group (180 ppm). Because rats (and humans with severe injury) do not consume nutrients orally in the first 24 h after injury, separate groups of animals fed the control and supplemented diets were also given intraperitoneal injections (i.p.) of zinc (30 mg/kg) 1 h after injury. As expected, TBI resulted in significant deficits in spatial learning and memory, as well as evidence of anxiety-like and depression-like behaviors. None of the zinc treatment regimens reduced anxiety-like behaviors in injured rats. However, treatment with dietary zinc supplementation alone was sufficient to significantly improve learning and memory. The combination of i.p. zinc followed by dietary zinc supplementation was required for significant reductions in depression-like behaviors [46], suggesting that early zinc administration may be beneficial in preventing TBI-associated mood disorders.

Investigators also tested the ability of zinc supplementation to enhance resilience to TBI. In this series of experiments, adult rats were fed a zinc-supplemented diet for four weeks prior to injury to test the hypothesis that chronic zinc supplementation would provide resilience to the cognitive and behavioral consequences of TBI [40]. Examination of spatial learning and memory beginning one week after TBI showed that zinc supplementation prevented cognitive deficits. In fact, throughout the 10 day Morris water maze test, zinc-supplemented animals were indistinguishable from sham operated controls, while untreated animals displayed significant impairments after TBI (*p* < 0.01). Similarly, while TBI induced significant anhedonia in the two-bottle saccharin test (*p* < 0.001), chronic zinc supplementation completely prevented the appearance of this depression-like behavior. This work showed that chronic intakes of zinc before injury provided more robust protection from deficits in cognitive and behavioral outcomes than that seen in experiments using zinc as a treatment [40] and points to the possible use of chronic zinc supplementation to prevent poor outcomes in populations at high risk for TBI.

### 4.3. Role of TBI and Zinc in Neurogenesis

While both the clinical and preclinical data collected to date suggest a possible role for zinc in the prevention and treatment of poor outcomes from TBI, the next steps in the continuum of research clearly need to be mechanistic in nature. One potentially fruitful avenue of research is the role of zinc supplementation in adult hippocampal neurogenesis. There are several lines of support for work in this direction. First, in vivo studies have shown that zinc is needed for normal adult hippocampal stem cell proliferation and neurogenesis [35,36,37,38,39]. This includes work showing that chelation of zinc specifically inhibits TBI-associated neurogenesis [43]. Secondly, newly born cells of the hippocampus have been implicated in the regulation of mood, antidepressant drug efficacy, and cognitive function [47,48,49,50]. Given that zinc supplementation has been shown to prevent depression and cognitive impairment associated with TBI, it was reasonable to hypothesize that zinc supplementation enhances neuronal precursor cell proliferation and neurogenesis in the hippocampus of injured animals.

To test this hypothesis, adult rats were fed a zinc-adequate (30 ppm) or zinc-supplemented (180 ppm) diet for 4 weeks followed by TBI using controlled cortical impact. Stereological counts of proliferating cells showed that TBI doubled the density of newly born cells in the dentate gyrus of the hippocampus 24 h after injury (*p* < 0.05). More importantly, supplemental zinc significantly increased this by an additional twofold (*p* < 0.0001). When the number of these cells surviving for one week were examined, the total density of newly born cells was still approximately 60% higher in supplemented rats [51]. Therefore, it appears that chronic dietary zinc supplementation has the ability to enhance cell proliferation in the hippocampus after cortical injury.

The next question was, do these cells go on to participate in adult neurogenesis, and if so, to what extent does zinc supplementation enhance the process of differentiation and maturation? Using doublecortin as a marker of neuronal differentiation, it was shown that regardless of diet or injury status 85% of newly born adult hippocampal neurons were doublecortin-positive after one week. However, because there were more newly born neurons in the chronic zinc supplementation group, the density of new doublecortin-positive neurons was higher at one week post-TBI. Furthermore, the zinc effect was shown to be maintained for at least four weeks after injury (*p* < 0.01). Use of targeted irradiation to eliminate the ability of zinc to enhance adult neurogenesis suggested that these cells, while not likely to be the only mechanism, appear to participate in the ability of chronic zinc supplementation to prevent the development of depression-like behaviors that are consistently associated with TBI [51].

## 5. Conclusions

**Figure 1:**
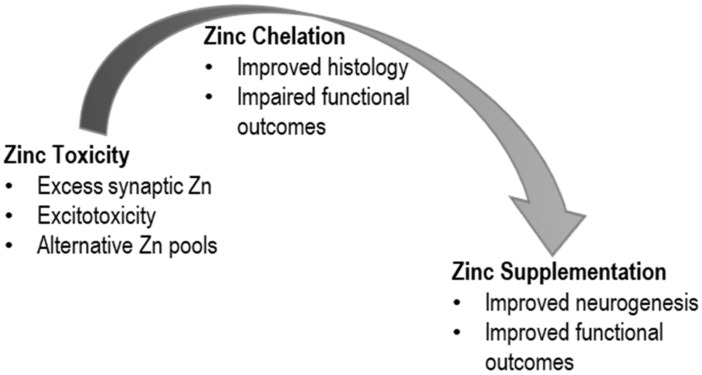
Research over the last three decades has followed a continuum that reflects shifting hypotheses about the role of zinc in traumatic brain injury. Initial work suggested a neurotoxic role for zinc that led to explorations into the use of zinc chelators. Subsequent work has suggested a beneficial role for zinc supplementation in improved outcomes associated with TBI.

Our understanding of the cellular, molecular, and behavioral roles of zinc in TBI is the result of a continuum of research spanning more than three decades (Figure 1). From initial landmark work on the neuronal toxicity of chelatable zinc after injury, the field expanded to include studies of zinc- and TBI-regulated gene expression, the role of zinc in neurogenesis, and the use of zinc to prevent poor outcomes associated with TBI. Future work along this continuum should include work to apply this wealth of information to treat patients with TBI in a clinical setting.
